# The Coronary Sinus Reducer; 5-year Dutch experience

**DOI:** 10.1007/s12471-020-01525-8

**Published:** 2020-12-07

**Authors:** M. J. M. Silvis, M. Dekker, C. Zivelonghi, P. Agostoni, P. R. Stella, P. A. Doevendans, D. P. V. de Kleijn, J. P. van Kuijk, G. E. Leenders, L. Timmers

**Affiliations:** 1 Department of Cardiology, University Medical Centre Utrecht, Utrecht, The Netherlands; 2 Department of Vascular Surgery, University Medical Centre Utrecht, Utrecht, The Netherlands; 3 Department of Cardiology, Amsterdam UMC, University of Amsterdam, Amsterdam Cardiovascular Sciences, Amsterdam, The Netherlands; 4 Hart Centrum, Ziekenhuis Netwerk Antwerpen (ZNA) Middelheim, Antwerpen, Belgium; 5 Netherlands Heart Institute, Utrecht, The Netherlands; 6 Central Military Hospital, Utrecht, University Medical Center Utrecht, Utrecht, The Netherlands; 7 Department of Cardiology, St. Antonius Hospital Nieuwegein, Nieuwegein, The Netherlands

**Keywords:** Refractory angina, Coronary artery disease, Coronary Sinus Reducer

## Abstract

**Background:**

Refractory angina is a growing and major health-care problem affecting millions of patients with coronary artery disease worldwide. The Coronary Sinus Reducer (CSR) is a device that may be considered for the relief of symptoms of refractory angina. It causes increased venous pressure leading to a dilatation of arterioles and reduced arterial vascular resistance in the sub-endocardium. This study describes the 5‑year Dutch experience regarding safety and efficacy of the CSR.

**Methods:**

One hundred and thirty-two patients with refractory angina were treated with the CSR. The primary efficacy endpoint of the study was Canadian Cardiovascular Society (CCS) class improvement between baseline and 6‑month follow-up. The primary safety endpoint was successful CSR implantation in the absence of any device-related events.

**Results:**

Eighty-five patients (67%) showed improvement of at least 1 CCS class and 43 patients (34%) of at least 2 classes. Mean CCS class improved from 3.17 ± 0.61 to 2.12 ± 1.07 after implantation (*P* < 0.001). The CSR was successfully implanted in 99% of the patients and only minor complications during implantation were reported.

**Conclusion:**

The CSR is a simple, safe, and effective option for most patients with refractory angina. However, approximately thirty percent of the patients showed no benefit after implantation. Future studies should focus on the exact underlying mechanisms of action and reasons for non-response to better identify patients that could benefit most from this therapy.

**Electronic supplementary material:**

The online version of this article (10.1007/s12471-020-01525-8) contains supplementary material, which is available to authorized users.

## What’s new?

- The Coronary Sinus Reducer is a safe, simple and effective option for the majority of patients with refractory angina
- The Coronary Sinus Reducer leads to a significant reduction of angina severity
- Our results support further research on determinants of effectiveness/ineffectiveness and long-term follow-up

## Introduction

Refractory angina (RA) is a growing and major health-care problem affecting millions of patients with coronary artery disease (CAD) worldwide [1]. RA is defined as long-lasting (for ≥3 months) symptoms that are caused by established myocardial ischaemia in the presence of obstructive CAD, without further pharmacological or revascularisation options [2]. It is estimated that up to 10% of patients with CAD have RA, and the incidence is expected to grow due to an ageing population and the increasing prevalence of severe and advanced CAD [3].

The quality of life of patients with RA is reduced significantly with debilitating symptoms and increasing hospitalisations. Additionally, RA is leading to an increase in related health-care costs [4, 5]. The number of potential therapeutic options that aim to improve symptoms and quality of life is rising, but large and thorough studies evaluating their efficacy and safety are limited [6, 7].

Currently, the European Society of Cardiology acknowledges that there are three non-pharmacological options to be considered for symptom relief in patients with debilitating RA despite optimal medical and revascularisation strategies: enhanced external counter pulsation, spinal cord stimulation and the Coronary Sinus Reducer (CSR) [2].

The CSR is an hourglass-shaped device that can be placed in the coronary sinus. The presumed mechanism of action is based on an increased venous pressure as a consequence of the CSR placement, which results in a dilatation of arterioles and reduced vascular resistance in the sub-endocardium. Consequently, the coronary blood flow is redistributed from the epicardium to the ischaemic subendocardial territory, leading to reduction of ischaemia and symptoms [8].

The first-in-men procedure was performed in 2005 and multiple studies have been published since then, showing that about 70–85% of patients receiving a CSR experience an improvement in symptoms [9–12]. The majority of these results are applicable to patients with an ejection fraction above 30% and objective evidence of reversible ischaemia affecting the left coronary territory, although some studies also mention positive effects on the right coronary territory [13]. Its efficacy is currently under study for several other indications, for example in patients without obstructive CAD, or with microvascular dysfunction. Additionally, growing evidence about the efficacy is collected from several multicentre, multinational registries [14–16]. Furthermore, recently the CSR was shown to decrease the health-care burden and related costs of patients with RA and it also appears to be cost-effective according to the Dutch societal willingness to pay threshold [17].

We aim to provide an overview of the safety and efficacy of the CSR in a real-world Dutch population. We will therefore present the results of the 5‑year Dutch experience regarding CSR implantation and provide recommendations on how to proceed with further research.

## Methods

### Study population

This is a multicentre study, investigating all patients who underwent a CSR implantation in the University Medical Centre Utrecht and the St. Antonius hospital in Nieuwegein between 2014 and 2020. Patients were eligible for CSR implantation if they suffered from symptomatic angina despite; (1) maximum tolerated pharmacological therapy, (2) no revascularisation options with percutaneous coronary intervention (PCI) or coronary artery bypass grafting (CABG) as decided by the local heart team, and (3) proven stress-induced myocardial ischaemia by non-invasive stress tests. Exclusion criteria were less robust when compared with the strict protocols for randomised controlled trials (RCTs) such as the COSIRA trial [11]. The most important exclusion criteria were: (1) successful revascularisation in the last 30 days, or (2) previous CRT device with a left ventricular lead. Informed consent was obtained from all participants.

### Data collection

Clinical data, e.g. symptoms at presentation, medical history (including medication and vital parameters), risk factors, information on hospital visits and information regarding the location and method of ischaemia detection were collected with the use of electronic patient files. Clinical follow-up regarding medication changes, hospital admissions and vital parameters were collected up to 6 months after CSR implantation. The primary endpoint of the study was Canadian Cardiovascular Society (CCS) class improvement as assessed by the treating cardiologist between baseline and 6‑month follow-up. Both any improvement, as well as the degree of improvement, were studied. A responder was defined as a patient with at least one CCS class improvement 6 months after CSR implantation. Most of the patients were also included in the Reducer‑1 registry for which an independent physician scored the CCS classification after 6 months. We used this CCS classification to determine our primary endpoint. Not all patients were included in this registry, however, regardless of their participation in the Reducer‑1 registry all patients were seen at the outpatient clinic 6 months after implantation. For these patients, we based our endpoint on the judgement of the treating physician.

### Device implantation

Implantation of the CSR was performed by an interventional cardiologist according to the previously outlined procedure, described in detail elsewhere [8]. To summarise, after local anaesthesia the right jugular vein was punctured, after which right atrial pressure was assessed. If this pressure was below 15 mm Hg the procedure was continued with fluoroscopic visualisation of the coronary sinus to evaluate whether the size was appropriate (diameter between 9 and 13 mm based on quantitative coronary analysis measurement or visual estimation). Once the patient’s coronary sinus was deemed suitable for implantation, the CSR was brought in position through a 9 Fr. guiding catheter. The CSR is delivered on a balloon which is inflated between 2–6 atmospheres for at least 60 s to reach approximately 15% oversizing of the coronary sinus. After successful implantation and closure of the access site, patients were discharged from hospital on the same day. Clopidogrel, in addition to aspirin or anticoagulation, was prescribed for 3 months. After 3 months, the pre-implant anticoagulation regimen was continued.

### Statistical analysis

Values were displayed as mean with standard deviation or as frequency with corresponding percentages. Baseline characteristics as well as disease characteristics were analysed for the total population and stratified on response. Differences in continuous variables were compared by independent t‑test or Mann-Whitney U test where appropriate. Dichotomous variables were compared with chi-squared test or Fisher’s exact test. Differences in mean CCS classification between baseline and 6‑month follow-up were tested with a paired Student’s t‑test. Supplemental analysis was performed with paired t‑test or Wilcoxon signed rank. All analyses were performed with IBM SPSS statistics version 25.0.0.2.

## Results

A total of 132 patients were eligible for CSR implantation. The baseline characteristics for all patients and stratified on response are shown in Tab. 1. Mean age was 66 years, 75.8% of all patients were male and an average BMI of 29 was observed. A history of coronary revascularisation was present in most patients (83.3% previous PCI and 76.5% CABG). The most frequently used antianginal agents were beta blockers and nitrates (both 77.3%). One hundred and eleven patients (84.1%) used 2 or more antianginal drugs and 58 (43.9%) used 3 or more. Stratified results revealed a higher number of current smokers among the responders (22.4% vs 2.6%, *P* = 0.006) and showed a higher percentage of patients using nitrates (84.7% vs 63.4%, *P* = 0.007). No differences were found with regard to age, sex or other traditional risk factors for CAD. Tab. 2 gives an overview of the type of disease among the patients. Up to 20% of patients had diffuse vessel disease, often indicating multiple affected coronary arteries without clear culprit lesions. Almost half of the patients had a chronic total occlusion (50% concerning the right coronary artery). The stratified analysis did not show any difference in disease characteristics between responders and non-responders. Procedural details are summarised in Tab. 3. In one case no CSR was placed due to the patient’s coronary sinus anatomy (too small) leading to successful placement in 99% of the patients. Mean procedure time was 37 (±22) minutes. Complications were seen in 6 cases. Most complications were dislocation of the device before it reached the target area. In all cases this could be solved, resulting in successful placement. In one patient multiple complications occurred (access site complication, wire perforation and dislocation of the device), but all complications were solved and the CSR was placed. None of the patients died during implantation or because of the procedural complications.

**Table 1:** Baseline characteristics

*n*	All (*n* = 132)	Non-responders (*n* = 41)	Responders (*n* = 85)	*P*-value
Age	66 (9)	66 (10)	67 (9)	
Male	100 (75.8%)	31 (75.6%)	65 (76.5%)	0.915
BMI	29.0 (6.8)	28.39 (3.83)	29.22 (7.94)	0.535
Ejection fraction	55 (10.6)	58 (9.0)	55 (11.0)	0.095
eGFR	68.47 (17.9)	65 (18.0)	71 (17)	0.064
*Risk factors*				
– Diabetes mellitus	58 (44.3%)	20 (48.8%)	34 (40.5%)	0.379
– Hypercholesterolaemia	75 (57.3%)	20 (48.8%)	51 (60.7%)	0.206
– Hypertension	96 (73.3%)	30 (73.2%)	61 (72.6%)	0.948
– Smoking (% current)	19 (16.1%)	1 (2.6%)	17 (22.4%)	0.006
– Family history CAD	42 (32.8%)	12 (30.8%)	29 (34.5%)	0.681
*Medical history*				
– Previous MI	83 (62.9%)	25 (61.0%)	52 (61.2%)	0.983
– Previous PCI	110 (83.3%)	37 (90.2%)	67 (78.8%)	0.318
– Previous CABG	101 (76.5%)	29 (70.7%)	67 (78.8%)	0.114
*Antianginal medication*				
– Nitrates	102 (77.3%)	26 (63.4%)	72 (84.7%)	0.007
– CCB	88 (66.7%)	29 (70.7%)	55 (64.7%)	0.501
– BB	102 (77.3%)	34 (82.9%)	63 (74.1%)	0.271
– Ivabradine	13 (9.8%)	4 (9.8%)	9 (10.6%)	0.886
– Ranolazine	1 (0.8%)	1 (2.4%)	–	–
– >2 antianginal agents	111 (84.1%)	34 (83.0%)	73 (85.9%)	0.976
– >3 antianginal agents	58 (43.9%)	17 (41.5%)	38 (44.7%)	0.622

Values are displayed as mean (SD) or frequency (%)

*BMI* body mass index, *eGFR* estimated glomerular filtration rate, *CAD* coronary artery disease, *MI* myocardial infarction, *PCI* percutaneous coronary intervention, *CABG* coronary artery bypass graft, *CCB* calcium channel blocker, *BB* beta blocker, *SD* standard deviation

**Table 2:** Disease characteristics

*n*	All (*n* = 132)	Non-responders (*n* = 41)	Responders (*n* = 85)	*P*-value
*Type of disease*				
– 0	1 (0.8%)	1 (2.5%)	–	–
– 1 vessel	41 (32.0%)	11 (27.5%)	28 (34.1%)	0.460
– 2 vessel	31 (24.2%)	7 (17.5%)	22 (26.8%)	0.256
– 3 vessel	26 (20.3%)	11 (27.5%)	14 (17.1%)	0.180
– diffuse	24 (18.8%)	7 (17.5%)	16 (19.5%)	0.790
– spasm	1 (0.8%)	–	1 (1.2%)	–
– microvascular	4 (3.1%)	3 (7.5%)	1 (1.2%)	0.067
*CTO*				
Presence Y/*N*	56 (48.3%)	13 (37.1%)	40 (53%)	0.146
*Ischaemia detection*				
Method of ischaemia detection				
– SPECT	97 (73.5%)	29 (70.7%)	63 (74.2%)	0.536
– Stress echocardiography	2 (1.4%)	1 (2.5%)	1 (1.2%)	0.608
– MRI	27 (20.5%)	11 (26.8%)	15 (17.6%)	0.260
– XECG	3 (2.3%)	–	3 (3.5%	–
– Unknown	3 (2.3%)	–	3 (3.5%)	–
*Location*				
– Anterior	53 (41.4%)	15 (36.6%)	33 (40.7%)	0.657
– Interior	59 (46.1%)	20 (48.8%)	35 (43.2%)	0.559
– Lateral	58 (45.3%)	16 (39.0%)	40 (49.4%)	0.278
– Apical	22 (17.2%)	8 (19.5%)	14 (17.3%)	0.762
– Septal	50 (39.1%)	19 (46.3%)	27 (33.3%)	0.161

Values are shown as frequencies (%)

*CTO* chronic total occlusion, *SPECT* single-photon emission computed tomography, *MRI* magnetic resonance imaging, *XECG* exercise electrocardiogram

**Table 3:** Procedural details

Procedures	Details
Time minutes (mean, SD)	37 (22)
Contrast ml (mean, SD)	57 (27)
Radiation mGy (mean, SD)	403 (317)
Access site complication	*n* = 2
Device embolisation	*n* = 2
Device dislocation	*n* = 3
Wire perforation	*n* = 1
Intraprocedural death	*n* = 0
Procedural tamponade	*n* = 0
Successful placement	*n* = 131 (99%)

*SD* standard deviation

**Fig. 1:**
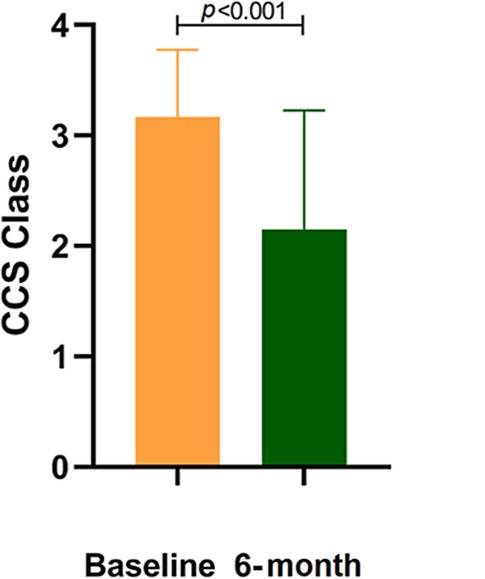
Comparison of mean CCS class. *CCS* Canadian Cardiovascular Society

**Fig. 2:**
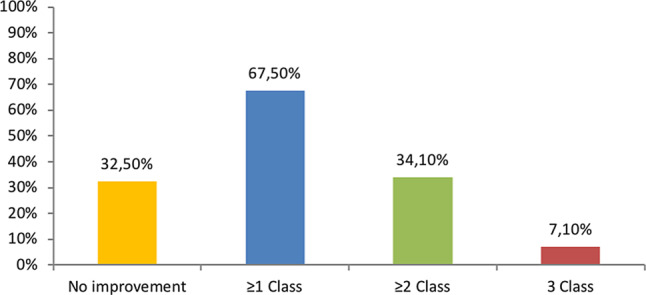
Amount of CCS class improvement after reducer implantation. *CCS* Canadian Cardiovascular Society

**Fig. 3:**
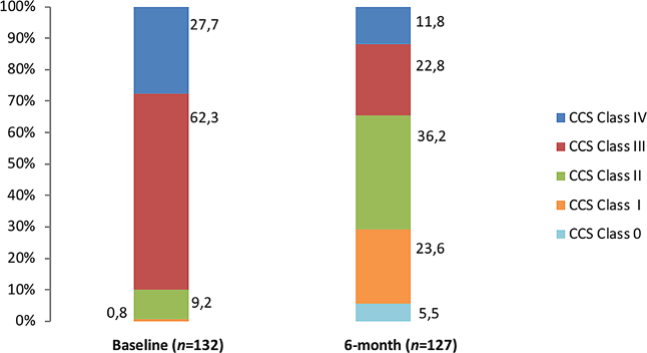
Distribution of CCS class at baseline and 6 months after reducer implantation. *CCS* Canadian Cardiovascular Society

Follow-up details were available in 127 patients. Treatment with the CSR resulted in a significantly lower CCS class after 6 months (2.12 ± 1.07 vs 3.17 ± 0.61, *P* < 0.001, Fig. 1). In total 67.5% of all patients improved at least one CCS class after implantation of the CSR (Fig. 2). Additionally, approximately one third of the patients improved 2 or more CCS classes and 7.1% 3 or more. Distribution of CCS class among patients is shown in Fig. 3. Before CSR implantation 90% of patients suffered from CCS class III and IV, after implantation this was reduced to 34.6%, corresponding to a reduction of 62%. Two patients received coronary revascularisation within 6 months after implantation (data not shown). Additional clinical follow-up details are summarised in Supplementary Tab. 1. Implantation of the CSR resulted in a significant reduction of hospitalisations as a result of anginal complaints (34.4% vs 11.7%, *P* < 0.001). The same was seen for visits at the emergency department (28% vs 15.8%, *P* = 0.009). No differences in blood pressure and heart rate were found during the 6 months before and after CSR (Supplementary Table 1).

## Discussion

In the current study, we show the 5-year experience regarding CSR implantation in a large, real-world Dutch cohort of RA patients. Within a time frame of 5 years, a total of 132 patients were included. A CSR was successfully implanted in 99% of patients and only minor complications during implantation were reported, which could be solved immediately. Furthermore, no long-term complications related to the device were observed.

CSR implantation significantly reduced the mean CCS class (3.17 before vs 2.15 after, *P* < 0.001) and an improvement of at least one CCS class at 6 months was seen in 67.5% of patients. Furthermore, we showed that at baseline a total of 90% of patients had complaints of debilitating angina at rest or with only mild exertion (CCS class III or IV). After implantation, this percentage decreased with 62% to only 34.6%, meaning more than half of the patients no longer suffered from severe complaints of angina. Additional analysis revealed a significant reduction in hospital admissions (34.4% vs 11.7%, *P* < 0.001) and visits to the emergency department (28.0% vs 11.7%, *P* = 0.009), which is in line with a recent study that reported decreased health-care resource use and related costs following CSR implantation [17]. Although medication changes after CSR implantation were allowed, these were very rare. No differences were found with regard to blood pressure and heart rate before and after the procedure, further indicating that the beneficial effects are indeed attributable to the CSR and not to changes in medical treatment.

The efficacy of the CSR is slightly lower than previously reported (70–85% compared with 68%), which could be explained by several reasons. Most importantly, we included patients with less robust inclusion criteria compared with the strict protocols that were used in previous studies. Another important difference with the existing RCT and registries is the lack of a uniform protocol. For example, we have included patients with an ejection fraction below 30% and patients with chronic renal failure. Careful evaluation before implantation is necessary in all patients and even more pivotal in patients with severe systolic heart failure that may need future cardiac resynchronisation therapy (CRT). Therefore, at this stage, CSR implantation is not advisable in patients with systolic heart failure with an ejection fraction <30%. However, two recent case reports suggest that CRT is still possible after the CSR is implanted, but further evaluation is needed [18, 19]. Moreover, we have also treated a few “no-option” patients with significant right-sided CAD and patients with significant CAD without objective ischaemia on SPECT or MRI. This could have affected our outcomes. However, we do believe that CSR treatment should be considered in all patients with RA not amenable to revascularisation and medication as currently stated in the ESC guidelines [2]. Furthermore, most patients in our study are patients referred for second or even third opinion, leading to re-evaluation of existing angiographic data and information regarding ischaemia. This automatically leads to a much more heterogeneous population compared with the previously performed RCT and registry studies in which patients were recruited by the treating physician [11, 12].

A clear explanation for non-response to this treatment is lacking, however, some explanations have been proposed. Firstly, the presumed mechanism of action is based on an increased venous pressure in the coronary sinus resulting in better oxygen supply of the ischaemic myocardium. This is based on the idea that in most patients the drainage system is relatively comparable. However, patients with an accessory venous drainage system could, therefore, benefit less from its working mechanism. A proposed measurement to evaluate the accessory venous drainage system is the differential pressure between baseline right atrial pressure and coronary sinus systolic pressuring during balloon occlusion of the CSR, with a low differential pressure indicating alternative drainage and thus potential non-response. Baldetti et al. showed a comparison of one patient with a high differential pressure and one patient without, which supported this hypothesis [20]. Other factors that have been proposed to play a causative role for non-responders are the presence of epicardial and/or microvascular spasm, symptoms unrelated to myocardial ischaemia (e.g. heart failure), incomplete endothelialisation of the CSR and inadequate pressure gradient across the device. However, robust data on these suggestions do not (yet) exist [8, 21, 22]. The lack of clear predictors for response and non-response is consistent with our findings as no differences were observed between baseline or disease characteristics, and treatment response.

### Future recommendations

Future studies should focus on the exact underlying mechanisms of action and reasons for non-response to better identify patients that could benefit the most from this therapy. Analyses of future CSR patients with objective ischaemia detection pre- and post-implantation are required to detect whether the beneficial effects could be objectified with an actual reduction of myocardial ischaemia. Additionally, it is necessary to evaluate the long-term efficacy and the effects in patients with solitary right-sided CAD. Finally, factors that have been proposed as causative factors for non-response (such as incomplete endothelialisation) should be evaluated on a larger scale.

### Limitations

The retrospective nature of our study is a clear limitation. Since this study was not performed in a controlled fashion, such as the previously performed sham-controlled COSIRA-trial, there was a lack of uniform data collection. Furthermore, the COSIRA-trial demonstrated a substantial improvement in angina symptoms in 42% of the sham-controlled group, indicating an important placebo effect in this population [11]. The primary endpoint was CCS classification assessed by the treating physician without objective measures of ischaemia, which due to its subjectivity is always ground for debate. Lastly, stratified analysis to identify differences between non-responders and responders should be interpreted with caution considering the small sample size.

## Conclusions

In this real-world, multicentre 5‑year Dutch experience, implantation of the CSR was shown to be a safe and effective treatment for the majority of patients with refractory angina. A clear explanation for the non-response in about thirty percent of the patients is still lacking. Our results support the need for further research investigating the determinants of effectiveness, ineffectiveness and long-term follow-up. In conclusion, a CSR is a simple and safe option for reducing symptoms in patients with RA in the Netherlands.

## Caption Electronic Supplementary Material

Additional clinical follow-up details (healthcare visits and vital parameters) are shown in supplementary table 1
