# Supplementary material for: The Coronary Sinus Reducer; 5-year Dutch experience
Source: Neth Heart J. 2020 Dec 7;29(4):215–23. doi: 10.1007/s12471-020-01525-8 (PMC7991026; doi:10.1007/s12471-020-01525-8)
Supplement: Supplementary file 1 — Additional clinical follow-up details (healthcare visits and vital parameters) are shown in supplementary table 1 [file 12471_2020_1525_MOESM1_ESM.docx]

**Supplementary materials**

| **Supplementary Table 1 Clinical follow-up** | | | |
| --- | --- | --- | --- |
|  | **6 months prior to CSR** | **6 months post CSR** | ***P* value** |
| **Healthcare visit** | *N = 125* | *N =120* |  |
| Hospitalisation* | 43 (34.4%) | 15 (11.7%) | <0.001 |
| ED visits | 35 (28.0%) | 19(15.8%) | 0.009 |
| OPC visits | 115 (92.7%) | 116 (96.7%) | 0.366 |
| **Vital parameters** |  |  |  |
| Systolic RR | 127 (16) | 128 (17) | 0.732 |
| Diastolic RR | 71 (17) | 73 (11) | 0.986 |
| Heart rate | 66 (9) | 67 (10) | 0.115 |
| **Hospitalisation for (un)stable angina. ED = Emergency Department, OPC = Outpatient Clinic* | | | |
